# Demystifying the “Victimized State”: A Civil–Military Crisis in Waiting?

**DOI:** 10.1177/1054137316675717

**Published:** 2016-10-22

**Authors:** Ross McGarry

**Affiliations:** 1 University of Liverpool, UK

**Keywords:** Chilcot inquiry, critical military studies, demilitarization, institutionalization, military mental health, stigma

## Abstract

The purpose of this article is to illustrate prescient issues relating to current and ex-military communities in the United Kingdom who have featured heavily within the policy arena over the past decade in relation to several key areas of importance. It will be illustrated how this population becomes visible within the public imagination (via military losses), how discourses relating to the harms they experience are structured and articulated within political and policy domains (particularly in relation to mental health) via “state talk” (qua Sim), and what the potential social consequences are for politically rendering an unproblematized populist view of current and ex-military communities (i.e., pending crises). This argument is made with the express intention of reengaging critical recognition of the distancing of the military institution from the physical and psychological vulnerability of those who have participated in war and military environments. This is an argument returned to pertinence from the recent publication of the Chilcot Inquiry into British involvement in the Iraq war.

## Introduction

As part of an international military coalition, the declared ‘original objective’ of the British New Labour government during the war in Iraq (2003–2009) was stated as removing Saddam Hussein from power; a proposed policy “success” intimated as conjoined with being ‘based on the *benefit of the target group*’ of the Iraqi population (McConnell, 2010, p. 107, *emphasis in original*). However, following the prolonged publication of the Chilcot Inquiry into British involvement in the Iraq war, the invasion and its aftermath has now been formally established as a foreign policy failure on behalf of the British government and Ministry of Defence (MoD; see Chilcot, 2016d, p. 109, para. 792). Although not making admissions of illegality within its remit, the Chilcot Inquiry documented that ‘the circumstances in which it was ultimately decided that there was a legal basis for UK participation were far from satisfactory’ (Chilcot, 2016e, p. 62, para. 432). While such statements perhaps offered anecdotal vindication to those who had mooted the grounds of this invasion as being illegal for over a decade (see inter alia, Kramer & Michalowski, 2006), commentators have suggested that Chilcot had not gone far enough in evidencing, for example, the industrial imperative for the enactment of war (Whyte & Muttitt, 2016). Also rendered marginal within the inquiry were the wider budgetary, social, and economic costs of this war, which have been estimated at three trillion dollars (see Stiglitz & Blimes, 2008). Crucially, however, ‘this number represents the cost *only to the United States*. It does not reflect the enormous cost to the rest of the world, or to Iraq’ (Stiglitz & Blimes, 2008, p. 31, *emphasis in original*). Within his opening address to the British Iraq war inquiry, Sir John Chilcot (2016d) did acknowledge that ‘the people of Iraq have suffered greatly’ (p. 9), as did other ministers responding to the inquiry’s findings. Again, however, such statements merely indicated—rather than reconnoitered—the lasting and deleterious costs of the war upon the “target group” of this dire failed UK foreign policy. In the war’s aftermath, the population of Iraq have had to adjust to a violent and ‘catastrophic normal’ (Iraq Body Count, 2016), evidenced from an estimated socioeconomic cost of 309 billion dollars to the country as a whole (Hagan, Kaiser, Rothenberg, Hanson, & Parker, 2012), over 250,000 civilian and combatant deaths caused from violence during the post-invasion phase to-date (Iraq Body Count, 2016), and ever-worsening circumstances to its eroding health infrastructure (World Health Organisation, 2016). Instead, more central to the inquiry’s criticism of the British establishment’s role in the Iraq war was the unnecessary risks which military personnel were said to have been placed under during their deployment from 2003 onwards. For Stiglitz and Blimes (2008), accounting for the consequences of such risks to military personnel is a central component for understanding the full domestic costs of the violence of the Iraq war. Such costs not only include current and future losses to the economy as a result of personnel being killed and injured, or expensive after care and pensions for military veterans being drawn from the public purse, it also includes untold ‘human costs’ affecting individuals, families, and communities (Stiglitz & Blimes, 2008, p. 18). However, a comprehensive view of the far-reaching costs of the Iraq war upon the serving and ex-military community in the United Kingdom also remained peripheral from the findings of the Chilcot Inquiry, and therefore public scrutiny.

In the wake of the 2003 Iraq invasion, and prior to the publication of the Chilcot Inquiry, the British Army did, however, lead the political agenda for the New Labour government, forcing them to respond positively to the needs of the military institution (Ingham, 2014). Over the past decade, the military doctrine drawn into the center of this debate was ADP Vol. 5: Soldiering: The Military Covenant, which outlines the expectations of British military personnel, how they should conduct themselves operationally, and what their core values should be. It also asserts the ‘unlimited liability’ they should be expected to commit to as a result of their service (i.e., losing their lives, suffering injury, etc.), a commitment that ‘must be sustained and provided for accordingly by the nation’ (Ministry of Defence, 2000, p. 1). Having been accused of “breaking” this Covenant, a litany of policy failures were leveled at New Labour throughout the Iraq war and its aftermath which, for McCartney (2010), included increasing military fatalities and a reemerging historical legacy of military veterans facing difficulties with their mental health. As British engagement throughout two unpopular wars in Afghanistan and Iraq continued to unfold, further controversies emerged exposing inept “operational welfare” for military personnel engaged in war (i.e., through a lack of kit, equipment, and training), insufficient awards of compensation for injured service personnel, lengthy delays into the inquests of service deaths in conflict, and demonstrations of overt public indignation toward service personnel in uniform (McCartney, 2010). Acknowledgment of these policy failures within the ministerial domain set in train a new “policy cycle” (qua McConnell, 2010) for electioneering political parties and the MoD.

In the lead up to the 2010 UK political elections, the military institution and its personnel became a prominent part of crossparty political agendas (Ingham, 2014). Having drawn attention to the failures of a “broken covenant” under New Labour, during 2008 former Conservative Party leader and British Prime Minister, David Cameron, established the Military Covenant Commission. A campaign founded on the tenets of the Military Covenant having been “broken” by the ‘cavalier manner’ of New Labour (Military Covenant Commission, 2008a, p. 5). Concerning itself with the provision of healthcare for military veterans and the welfare of serving personnel and their families, the Commission launched two flagship documents. The first addressing the “ill health” of the Military Covenant via deficiencies between civil–military and governmental relations with the Armed Forces (Military Covenant Commission, 2008b). The second proposing recommendations to “restore” the Military Covenant, tackling areas such as civil–military social cohesion, and “operational welfare” (Military Covenant Commission, 2008c). In tandem with the Commission’s campaign, and as noted in the Chilcot Inquiry (Chilcot, 2016b, para. 181–191), during 2007 New Labour launched an independent inquiry into the relationship between the Armed Forces and society (see Davies, Clark, & Sharp, 2008)*.* In its response, within the National Recognition of “Our” Armed Forces Report (Ministry of Defence, 2008), recommendations were made to host more civil–military public engagement events and offer improved health service provision, employment opportunities, and increased charitable support for military veterans. In doing so, a gateway was opened for formal policy implementation that symbolized the “mending” of policy failure with regard to the Military Covenant, setting the current context of civil–military relations in the United Kingdom. A resultant flagship document emerged in the form of the Armed Forces Covenant, a distinct policy that has been frequently misread as the Military Covenant and has since become the centrifuge for initiatives fostering civil–military relations within the United Kingdom such as the Community Covenant, Corporate Covenant, Armed Forces Day, and the recent Veterans Universal Passport (see Palmer, 2016).

However, unresolved policy failures that prevail from the 2003 invasion of Iraq have recently reemerged within the ministerial domain following the prolonged publication of the Chilcot Inquiry. Similar to the analysis raised by Stiglitz and Blimes (2008) regarding the U.S. military, part of these failure include a number of the 179 British military deaths and countless injuries occurring as a result of being accountable to the New Labour government failing the “operational welfare” of military personnel when embarking upon war (Chilcot, 2016a; McGarry, Mythen, & Walklate, 2012). Moreover, the Chilcot Inquiry recognized the cumulative impact of military deployments breaching ‘harmony guidelines’ (deploying for over 13 months within a 3-year period) and increasing the risk of posttraumatic stress disorder (PTSD) in serving personnel (Chilcot, 2016c, p. 66, para. 140). Set against a backdrop of the British civil–military policy landscape outlined earlier, the aftermath of the Chilcot Inquiry’s findings provide an opportune moment to critically reflect upon the social and cultural costs of this war’s violence (qua Stiglitz & Blimes, 2008) that are likely to have endured within the UK civil–military policy process.

This article will illustrate how these issues are made visible, or rendered less prominent, within the public and ministerial domains. It will do so by not only highlighting military losses at war but how discourses relating to the harms experienced by current and ex-members of the British military are structured and articulated within academic and policy research (particularly in relation to mental health). Critically, this article will also draw attention to what the potential consequences are for constructing an unproblematized view of these communities within civil–military policy making. To make this argument, Joe Sim's (2004) conceptualization of the “victimized state” will be theoretically and conceptually developed to interrogate the institutional and institutionalized harms of current and ex-military personnel by pursuing the following lines of inquiry: (a) what “losses” are made visible, (b) how is “illness” governed, and (c) what potential “crisis” is being obscured from view. The express intention of the following discussion is to stimulate a critical debate relating to the physical, psychological, and sociocultural costs of military service, and the ways in which these harms are frequently disassociated from the institutional environment of the military.

## Imagining the “Victimized State”

Joe Sim’s (2004) concept of the “victimized state” provides a critical engagement with state harms relating to criminal justice institutions. Drawing out two key features of his analysis, we are first informed of the particular types of interpersonal violence publicized as being experienced by state “servants” (i.e., prison and police officers) as a normative part of their employment. Second, the ways in which official discourses are constructed to report upon the occurrence of such violence is brought to our attention to indicate what other institutional harms are being obscured from view. For clarity, brief explanations of these concepts are each provided in turn.

### The Normalization of Violence: The “State Servant” as Victim

In drawing our attention to the ways in which knowledge is produced and constructed regarding the violence and vulnerability of criminal justice institutions and their “servants,” Sim (2004) avers that,

the symbolic position that prison and police officers command, have allowed the (*Prison Officers*) association to construct a clear and precise definition of the ‘truth’ around the normalised nature of prison violence and the risks and dangers faced by its members. The ideological power of this ‘truth’, which reproduces and reinforces the broader populism concerning the difficult job that state servants do, is such that alternative definitions of social reality remain subjugated and muted. (p. 117, *my insert*)

As such, criminal justice authorities construct knowledge and cultivate representation regarding the nature of violence experienced and enacted by those who serve as members of state institutions. For Sim (2004), the “truth” crafted around the vulnerability of “state servants” promotes an image of the “victimized state” and obfuscates the enactment and prevalence of institutional or institutionalized violence. Unpacking this further, Sim (2004) brings to our attention that the types of violence encountered by “state servants” (i.e., death and injury) are often misconstrued as disproportionately individualized instances of interpersonal violence rather than products of their structural environment (i.e., ill health, safety, etc.). The violence experienced by “state servants” is exclusively constructed as being received from the “enemy” rather than the structural and occupational conditions of prison work or policing (Sim, 2004). When deaths do occur to “state servants,” however, they carry heavy symbolic weight and serve to represent ‘a potent symbol of lawlessness’ (Sim, 2004, p. 121).

### “State Talk” and the Construction of Official Discourse

Next, drawing upon the work of Corrigan and Sayer (1985), Sim (2004) mobilizes the term *state talk* within his analysis as a way of illustrating what dangers to the “guardians of social order” look like, what the consequences for them are, and how they should be corrected and responded to by civil institutions and society. For Sim (2004), “state talk” within the arena of criminal justice becomes a means of recognizing the communication of harms experienced by the “victimized state” to the public, serving to reestablish normative perceptions of social and moral order. Through an interrogation of publicly available data, Sim (2004) demonstrates how those harmed when engaged in prison work and policing frequently suffer from accidents and ill health induced by the institutional environments of the workplace, rather than more commonly known instances of rare interpersonal violence. As such, public attention is drawn to individualized symbols of the “victimized state” and a “few bad apples” operating within in it. The institutional and institutionalized violence of the prison and police settings thereby become obscured (or “mystified”) from view, and only narrowly observes further ill treatment and harm enacted upon those in the “care of the state” (i.e., the public; Sim, 2004). The value and authenticity of this strand of Sim’s (2004) analysis is evident from its direct critical engagement with theory, policy, and the construction of prevailing state-centric discourse.

The argument developed by Sim (2004) provides a theoretical basis for critically understanding the political and public image of violent state institutions, and the harms experienced, and perpetrated by, their employees. However, there are absences within this analysis that limit its application to other contexts. By only addressing the criminal justice system, the theoretical concept of the “victimized state” falls short of a more thoroughgoing analysis of other violent institutional environments and policy landscapes.

## Conceptualizing “Loss” and “Illness” of the “Victimized State”

In view of these observations, within this and the forthcoming section, Sim’s (2004) “victimized state” is offered some conceptual and theoretical reconfiguration to see what his analysis would look like when applied to the contexts of the military and the MoD. To do so, a different literature and evidence base are drawn upon to that of Sim (2004) to extend critical scrutiny from the criminal justice landscape, to debates and contexts attendant to military sociology and critical military studies (see Basham, Belkin, & Gifkins, 2015). This is achieved by problematizing what is obscured from view via the construction of two codependent political and policy narratives: the first relating military deaths and the second to military mental health. First, however, both of these narratives are offered a brief outline before attention is turned to how they can be better understood as matters of the “victimized state.”

### Military Deaths on the High Street

The duration of the war in Iraq resulted in 179 British military deaths between 2003 and 2009, with a further 456 having perished in Afghanistan between 2001 and 2015. Although aware of the occurrence of these deaths via announcements from the MoD, news reporting, and fastidious documenting by BBC News (2016a, 2016b), the visual presence of military fatalities were thrust into the public imagination via events in the Wiltshire town of Royal Wootton Bassett between 2007 and 2011. Here, 167 individual military repatriation processions passed through the high street of Royal Wootton Bassett carrying the bodies of 345 British military personnel, as they were transported from a nearby airbase (RAF Lyneham) to a local coroner (see McGarry, 2016). For some, such as regularly attending townsfolk, these events were engaged with as nonpolitical acts of respect and public mourning (Walklate, Mythen, & McGarry, 2015). For others, however, this regular presence of military fatalities within the public domain represented political co-option to “mend” the “broken covenant” of UK civil–military relations and foster legitimacy for the wars in Iraq and Afghanistan (Jenkings, Megoran, Woodward, & Bos, 2012). The high visibility of military losses within the public domain can consequently be understood as attempting to politically obscure many deaths having occurred from occupational negligence at war, as noted by Chilcot (2016a), rather than from interpersonal “enemy” violence. Such accidents, as Hicks (1993) suggests, while perhaps routine and unintentional are found by the public and the military institution to be ‘even more horrifying than the more ordinary, enemy-inflicted variety’ (p. 389).

However, in outlining this first narrative, the situating of Sim’s (2004) analysis requires some reconceptualization. As Helen McCartney (2011) observes, British male soldiers (particularly during WWI and II) have preoccupied the public imagination and academic research as both delinquent young offenders (see inter alia, Spencer, 1954) and as “victims” (see inter alia McGarry & Walklate, 2011). Both are suggested as carrying warnings for the reputational damage to the MoD. However, contra Sim (2004), it is the construction, representation, and illustration of British soldiers as vulnerable subjects that is said to hold the greatest threat, rather than augmentation, to civil–military policy and the military institution. McCartney (2011) notes that,

the rise of this “victim” image could impact on the Army’s recruitment and retention, duty of care issues and the long-term public view on the future size and legitimacy of the Army itself. All these issues have serious implications for the formulation and execution of defense policy under the current coalition government and beyond. (p. 43)

In contrast to Jenkings et al. (2012), civil–military policy formation, as outlined within the introduction to this article, is suggested as serving to intentionally mitigate a “soldier–victim” image and reduce a continuum of institutional and social consequences (McCartney, 2011). As such, while military deaths on the high street of Royal Wootton Bassett may be understood to operate in the protective interests of the military in ways similar to criminal justice institutions (qua Sim, 2004), for the MoD, it instead holds significant threats to the reproduction of military interests and imperatives. What this brings to our attention is not only the existence of military deaths from engaging in war, and the further potential vulnerabilities to be experienced by service personnel, but a ministerial requirement for these issues to be closely managed as a result.

### Military Mental Health Research During the War in Iraq

As an extension of the high profile deaths experienced by military personnel in the wars in Afghanistan and Iraq, it is the impairment to military veterans’ mental health and welfare that has led headlines and policy since the beginning of the war in Iraq. As noted by the House of Commons Defence Committee (2008, Ev 69, *my insert*), a specific institution captures research of this nature regarding current and ex-military personnel,

This does not seem to get much publicity, but, of course, we (*the Ministry of Defence*) have a major contract with King’s College in which we are monitoring and assessing those people who served in Iraq and Afghanistan on a variety of issues.

Following an unsuccessful class action mounted against the MoD for failing to adequately treat psychological ill health among military personnel in the aftermath of the 1991 Gulf War, King’s Centre for Military Health Research (KCMHR) were commissioned with the MoD contract for conducting research on the mental and social well-being of current and ex-military communities (Green, 2015). As the major contract holders of the MoD, KCMHR is instrumental in informing military policy and political debate regarding the mental welfare of the UK Armed Forces. It is not the intention to explicate the sizeable back-catalogue of this work here. However, turning to the Chilcot Inquiry (2016c), it is possible to provide a brief insight into its contours.

Citing three key papers reporting on a major cohort study from KCMHR (see Forbes, Fear, Iversen, & Dandeker, 2011; Horn et al., 2006; Hotopf et al., 2006), Chilcot (2016c) draws attention to the main tenor of these findings. For Hotopf et al. (2006), there were no apparent links between participation in the Iraq war and poor mental health to Regular British military personnel at the time of their research (Phase 1), although significant effects were evidenced in Reservist personnel. Concurrently, in contrast to the aftermath of the 1991 Gulf war, Horn et al. (2006) found no “substantial increase” in the mental health of military personnel who had participated in the war in Iraq and those that had not. Later research from Forbes et al. (2011, p. 18) reiterated these findings (Phase 2), adding that the extent of mental health disorders for military personnel having served in Iraq and Afghanistan remained “low,” with PTSD in particular being experienced by a ‘relatively small proportion of military personnel.’ It was, however, noted that concerns remained with regard to high alcohol misuse in Regular military personnel and the mental health of combat troops and Reservists (Forbes et al., 2011). As Chilcot (2016c) outlines from the finer detail of these studies, there was deemed to be no increase in mental health problems for Regular military personnel regardless of service in Iraq or Afghanistan. PTSD was experienced at a slightly higher rate than the civilian population (3%) for those who had deployed to either theatre (1.3%–4.8%), and roughly the same rate as the civilian population for common mental disorders (i.e., depression and anxiety; Chilcot, 2016c, p. 66, para. 140). More pressing issues were reported to include alcohol misuse from across the Regular military population and higher “persistent” rates of PTSD in Reservists (Chilcot, 2016c, p. 66, para. 140). Although uncited by Chilcot (2016c), this evidence was supported by Fear et al. (2010) who maintain,

Our main finding is that, overall, the prevalence of mental disorders in the UK armed forces remained stable between 2003 and 2009. For regular personnel, we did not detect an effect of deployment to Iraq or Afghanistan on two of the three outcomes (probable post-traumatic stress disorder and common mental disorders) but we did record a modest effect of deployment on alcohol consumption. (p. 1792)

KCMHR (2014) later truncated these findings, restating that ‘As yet, there is no evidence of a ‘bow wave’, ‘tidal wave’ or ‘tsunami’ of mental health problems in UK Regulars or Reservists’ (p. 1).

### “State Talk” and an Orthodox “Civil–Military Nexus”

Having outlined both narratives of military “losses” and “illness,” how can Sim’s (2004) notion of the “victimized state” help us to critically consider the military institution and the MoD? There are (at least) two answers to this question. The first answer relates to recognition of the threat that public displays of military losses can pose for the operational equilibrium of the British military establishment and MoD. Considered in relation to the policy context of the Armed Forces Covenant outlined in the introduction to this article, and the policy tension relating to the “victim–soldier” image (qua McCartney, 2011), military personnel should be understood as occupying dual roles: They are both “state servants” (as per the Military Covenant) and in the “care of the state” (as per the Armed Forces Covenant). As such, in the aftermath of the Chilcot Inquiry (Chilcot, 2016c), scrutiny regarding the deaths and injuries of military “state servants” harmed by the “enemy” and other means during the Iraq war are likely to return attention back upon the military institution and its (mal)practice.

The second answer is found in a capacity to critically identify “state talk,” the official channels from which it derives, and what it is serving to maintain. By juxtaposing the narratives given earlier, we learn that a fundamental aspect of protecting the identity of the military institution (as *not* being vulnerable or problematic) is managed through the construction of official discourse relating to the harms its personnel experience as normative aspects of their employment. Therefore, discourse regarding the potential vulnerabilities of this population needs considering as being moderated from the subscribed institutional “state talk” of the MoD. Keen to make stark contrasts with past military failings in relation to the aftermath of the 1991 Gulf war, Horn et al. (2006) proposed, ‘Our results will serve to ally concerns raised by sporadic reports of a new Iraq war syndrome, or Gulf war syndrome II’ (p. 1745). They conclude,

It is possible that one factor that amplified, even if it did not create, the Gulf war syndrome crisis, was the perceived neglect of health surveillance and research on both sides of the Atlantic, allowing rumour and conjecture to flourish. The implementation of improved health surveillance, including but not restricted to the present study, might have also reduced some health concerns. (p. 1746)

The tone of this research provides an indication of how “state talk” has been operationalized since the beginning of the Iraq war to construct a counter-narrative to military vulnerability. Having learned lessons from the 1991 Gulf war, we come to understand that closer control of “improved health surveillance” for the MoD becomes a key technology to managing how vulnerability is reported to, perceived, and understood by the public and popular press. From more recent findings, we are left with similar key headlines defined by “state talk”: poor mental health is generally not a problem for military personnel, PTSD and common mental disorders are experienced in marginal and unremarkable ways in relation to the civilian population (see also Jones, Rona, Hooper, & Wessley, 2006), and alcohol misuse, depression, and anxiety are more common and prevalent concerns (see also Iversen & Greenberg, 2009).

Research of this nature is not novel to the British military or the contemporary period to which we are referring here, however. As an academic descendant of the sociopsychological work of Stouffer, Suchman, DeVinney, Star, and Williams (1949) in The American Soldier, the study of the mental health of current and ex-military personnel within past and present research is captured by military sociology. Evident in the research outlined earlier, it has at its core an investment in nomothetic, functionalist knowledge regarding the military institution and its personnel, wherein there is a specific paradigmatic preference for how harm should be conceptualized (Coates & Pelligrin, 1965). Put bluntly, ‘it should be based on sound statistics, not catchy sound bites’ (Holmes, Fear, Harrison, Sharpley, & Wessely, 2013, p. 2). For Jenkings et al. (2011), it is this enclave of an orthodox “civil–military nexus” that requires further critical scrutiny. It is a setting in which “top-down” research is produced by partnerships between military and academic institutions, wherein knowledge relating to the current and ex-military population is constructed as a ‘product of its political as well as intellectual context’ (Jenkings et al., 2011, p. 40). Woodward and Jenkings (2011) aver that within sociological military research of this nature, the experiences of military identities are thereby something to be ‘mapped, described and measured’ in an attempt to quantify and model behavior in ‘the pursuit of military objectives’ (Woodward & Jenkings, 2011, p. 254). As such, following more than a decade of research from the MoD, and despite evidence from the United States suggesting otherwise (see inter alia Hoge & Castro, 2006; Hoge et al., 2004), we are informed that there is nothing particularly exceptional about the occupational impacts of military service, nor the experience of operational environments or war. Taking stock of the narratives outlined earlier, this discussion now turns to consider what the reconstruction of the “non-victimized state” has the potential to be obscuring from view.

## The “Mystification of Harm”: Putting Distance Between Problems and the Institution

It is evident from previous research that the structural qualities of service life may indeed be protective and stabilizing (see also Bouffard & Laub, 2011; Sampson & Laub, 1996) and most ex-military personnel have seemingly unproblematic transitions to civilian life. However, the scepticism which underpins the present discussion resides in the degree to which this has become *the* prevailing view of current and ex-military communities at the expense of critical alternatives. The previous section has well illustrated the controlled construction of “loss” and “illness” as experienced by these populations, yet critical claims that run counter to the research established earlier are frequently asserted as untrue, indeed “mythical,”

Evidence from younger veterans is that the majority transition into civilian life without significant difficulties. However, many myths have been perpetuated by the media and others, including assertions that military veterans are more likely to take their own lives compared with others in society, a greater proportion have mental health problems, are in prison, or are sleeping rough on a regular basis. None of these myths are true. (Leach, 2016, *online*)

This perspective has become a popular trope supplanted within policy and research at the apex of a “civil–military nexus” (qua Jenkings et al., 2011), concerning the mental and social well-being of current and ex-military communities (see for example, Kelly, 2014, p. 2; Phillips, 2014, p. 1). It was also perpetuated in evidence within Section 16.2 of the Chilcot Inquiry (Chilcot, 2016c, p. 72, para. 162) whereby a “top-down” view of military mental health and welfare is covered comprehensively, as is the attendant support for ex-military personnel. But what is notably absent from the inquiry is a critique of the impacts of the military institutional environment upon the health and well-being of service personnel and military veterans. For example, as a singular comment toward the end of Section 16.2, evidence from the highest ranking medical officer in the British military, Lieutenant General Lillywhite (2010), acknowledges that stigma relating to mental health in the military remains a ‘perennial challenge’ (Chilcot, 2016c, p. 68, para. 143). However, despite its continuance within the military and ability to inhibit help-seeking behavior, as with other military mental health research outlined earlier, it is suggested to be a collective problem of the social, not just the military (Lillywhite, 2010, pp. 55–56). The distancing tenor of this comment becomes evident elsewhere. For example, as KCMHR (2014) report, ‘Stigma remains a barrier to help-seeking for serving and ex-Service personnel. There is no evidence that stigma is worse because of a Service background’ (p. 4).

Similarly, with regard to substance misuse and “poor behavior” postservice, recent findings from Kiernan, Arthur, Repper, Mukhuty, and Fear (2015) suggest that these characteristics are common within Army infantry personnel. They are, however, keen to conclude that ‘the assumption that the British Army infantry is, in itself, a cause of these behaviours should be questioned’ (Kiernan et al., 2015, p. 1). Considered alongside previous research from KCMHR (2014), which added suicide for military personnel was no worse than experienced within the civilian population, a consistent message is propounded: postservice difficulties are not only marginal but experienced by a maligned few, discharged from service among an unproblematic (or “heroic”) majority considered to have made the transition from military to civilian life “successfully.” From the “top-down” view of military sociological research noted earlier, we can surmise that the measurement of such “success” is made on the basis of ex-military personnel not having been immediately or latently captured by mental health or criminal justice statistics. An underlying problem with this approach, however, is the assumption that those who need help seek it, and that the psychological and social costs of military service and war are only narrowly defined by the nomothetic view of the “civil–military nexus.” More importantly, the cultural environment of the military institution is consistently neutralized from view.

When situated in the context of military personnel being not only “state servants” (qua Sim, 2004) but also in the “care of the state,” asserted here is the potential psychological and social problems they may face being frequently disassociated from the military institutional environment as a “mystification of harm.” In response to these observations, within the final section of this discussion, a challenge is made to the influential discourses of “state talk” to indicate what is perhaps being obscured from view by “state talk” and “top-down” approaches to military health research.

## Demystification: Bringing the Military Institution Back Into View

For Ruth Jolly (1996), experiencing war, disconnecting from civilian perspectives, or remaining in contact with nonmilitarized environments, all engenders unpredictable outcomes for ex-military personnel. Akin to these observations, some 20 years later, Wainwright, McDonnell, Lennox, Shaw, and Senior (2016, p. 12) give more recent appreciation to the influence of the military institution upon its personnel. They found that ingratiating people in militarized cultures creates “dependencies” and “idiosyncrasies” for its personnel and “associated dysfunctional behaviours” that are often discordant with some aspects of civilian life (such as alcohol misuse, exposure to violence, and machismo). With these more “bottom-up” observations in view (qua Jenkings et al., 2012), the final remarks made here advocate for reasserting the institutional context of the military back into the discourse of prevailing “state talk” regarding the mental and social well-being of current and ex-military communities.

### From Within: Common Mental Disorders, Stigma, and Suicide

As noted by Wainwright et al. (2016), such “dependencies” and “idiosyncrasies” of service life (i.e., routinized living, provision of amenities, and resilience) do not always have positive outcomes. Reviewing the same cohort study data cited by Chilcot (2016c), recent research from Goodwin et al. (2015) observed that the prevalence of common mental disorders were more than double for the occupational group of military populations than the general working population in the United Kingdom. As associations were not found to be linked to previous deployment or when stratified by demographic characteristics (i.e., age, gender, and education), they conclude that ‘it is therefore difficult to explain why the prevalence found in the military study is so much higher than the general population’ (Goodwin et al., 2015, p. 8). Their reasoning as to why this might be the case includes external factors to the military (i.e., childhood adversity and socioeconomic class), methodological and sampling issues, and problems of disclosure (Goodwin et al., 2015). Other research has reported that ex-military personnel experience a higher prevalence of suicide in younger service leavers than their civilian counterparts (Kapur, While, Blatchley, Bray, & Harrison, 2009). However, although mooting the possibility that “in-service exposure” to adverse experiences may have an influence on this population, Kapur et al. (2009) concluded that this was not their intention and were therefore ‘unable to draw any firm conclusions about the importance of these factors’ (p. 0007), without defining what such “factors” might be. Stated instead is the possibility that the military may be a *protective* environment for suicide prevention, rather than a damaging one. Once again, although suggested by Goodwin et al. (2015) as an area requiring further investigation, what is not given due consideration in these more recent studies are ‘specific aspects of military life’ (p. 10).

In a later response, Sareen and Belik (2009) advocated that what required further critical attention from Kapur et al.’s (2009) findings were those who had committed suicide as being less likely to have presented to mental health services the week before their deaths. The explanation of this from Kapur et al. (2009) rested on the possible difficulties for military veterans accessing civilian health services. However, Sareen and Belik (2009) instead urge for other sociological explanations to explore the possibilities of ex-military personnel feeling disconnected from society as a symptom of their disengagement from support services. Other studies have since been active in this regard in addressing the stigma of mental health within the military institution. For example, as McGarry, Walklate, and Mythen (2015) aver, the inculcation into military life is one that is both institutionally functional during service but also fosters an environment of hypermasculinity that stigmatizes mental health as weakness, fosters negative attitudes to help-seeking behavior (Brunger, Serrato, & Ogden, 2013; Hoge et al., 2004; Iversen et al., 2011; Sharp et al., 2016; Lincoln, Ames, & Moore, 2016), and encourages unhealthy relationships with alcohol use (Iversen & Greenberg, 2009). Once learned, these behaviors are not easily unpracticed and are unlikely to simply disappear upon discharge from the military, including for those who may never come under the nomothetic gaze of state services (McGarry et al., 2015). For Lincoln et al. (2016), capturing these experiences instead requires an engagement with culturally responsive qualitative research that is out of the reach of ‘the neutral, obfuscating language of official policy’ (p. 7). The argument being developed here follows this rationale and advocates for more research of this nature to illuminate an awareness of institutionalized vulnerability existing on a continuum, not in isolation, of merely being a military “veteran.” It is to this final problem that this discussion now turns its attention.

### From the Inside to the Outside: Institutionalization and Demilitarization

While institutionalization is perhaps not an “irreversible” process (qua Berger & Luckman, 1966), Jolly (1996) avers that what is consistent for institutionalized service personnel is the unifying experience of individual identities having been militarized, and the need to renegotiate this upon returning to civilian life, ‘for the military incorporates the *identities* of its members, and when they leave, they have somehow to rediscover themselves as separate, self-motivating, vulnerable individuals’ (p. 38, *emphasis in original*).

The experience of institutionalization as an occupational necessity of military employment should therefore not be overlooked in future research on the military, even for those deemed as making “successful” transitions from military to civilian life. Participants from Lincoln et al.’s (2016) findings illustrate that becoming a militarized person requires a mastery of drills and competencies that are not easily unlearned when discharged from the military. Reminiscent of Schuetz’s (1945) “homecomer,” when returning to civilian environments from military service, a “new normal” has to be recognized as needing to be adapted to (Lincoln et al., 2016). For example, it has recently been evidenced that the militarized “idiosyncrasies” of ex-service personnel are often more transferable within other institutionalized environments such as prison (Logan & Pare, in press; May, Stives, Wells, & Wood, 2016). However, due to the normativity of some behaviors enacted during service (i.e., sleeplessness, hyper-vigilance, etc.), and an unfamiliarity with some aspects of the throes of regular civilian living when returning home, these may not be immediately recognizable as indicators of problematic institutionalized behavior (Lincoln et al., 2016). Although institutionalized military behavior may not be ever-present for all current and ex-service personnel, ‘neither is it superficial. A threshold has been crossed’ (Jolly, 1996, p. 37). As a participant from Lincoln et al.’s (2016) findings acutely corroborates, ‘You don’t just get rid of it because it’s—you’re so in tune with it. It’s taken years and years and years and years to get to that level. You’ve honed it. You’ve perfected it’ (p. 6).

This is not to suggest that the transition from military to civilian life is a neglected aspect of service experience, however. British military personnel have recently been issued with A Welfare Guide for the Service Leaver (British Army, 2013) and attracted attention from Lord Ashcroft’s (2014) The Veterans’ Transition Review. Although addressing the role of the military institution in aiding military resettlement, as with research from KCMHR and the Chilcot Inquiry, what is missing from this additional body of “top-down” knowledge is the underpinning influence of military culture on those who are reentering civilian life. What this further obfuscates is a necessity for “institutionalized roles” (i.e., serving in the military) requiring its reenactment within social life to ensure institutional survival (Berger & Luckman, 1966). The performing of “institutionalized conduct” (i.e., militarized behavior) from the inside to the outside, reenacted through given institutional “roles” (i.e., being a military veteran), maintains the presence and legitimacy of influential social institutions, such as the military (Berger & Luckman, 1966). As such, it is argued here that reestablishing the connections between the cultural influences of the military institutional environment and civilian life is of key importance to offer critical rebuttals of institutional and ministerial “state talk.”

## Conclusion: A Crisis in Waiting?

The central concerns within this article have not only been targeted toward the increased visibility of the British Armed Forces within the public domain nor the growing presence of this occupational group within social policy. This article has instead been concerned with the conceptual forces underpinning what we are permitted to know about the hardships and experiences of this population when communicated through nomothetic state-centric research and, most importantly, what this has the potential to withhold from scrutiny. By illustrating how institutionalized vulnerability can be obfuscated from public view in the interests of the military institution, this article concludes by following Brown (2008) in proposing that current policy making and research regarding serving and ex-military communities to be potentially facilitating a crisis in waiting. As KCMHR suggest, the mental health, well-being, and misuse of alcohol within current and ex-military populations should remain a concern for future research. However, these, and other issues, should only be considered marginal when based on multiple sources of evidence that are considerate of both quantitative and qualitative approaches to critical studies of the military. “State talking” from official domains such as the MoD should not be permitted to “mystify” from view the influential role of the military institution as part of the potential problems faced by current and ex-military personnel. Moreover, we should be continually motivated to engage with the “bottom-up” qualitative experiences of current and ex-military communities and be ready to reconsider not only well-established problems (i.e., alcohol and drug misuse, crime) and those deemed as marginal (i.e., mental and emotional health, suicide, and homelessness). We must also be prepared to fully explore arenas that remain on-going (i.e., stigma) and those that are underexplored geopolitical elements of civil–military interaction (i.e., age, sexuality, class, gender, ethnicity, and religiosity; see inter alia Basham, 2013; Ware, 2012). Finally, we must be willing to consider the experience of demilitarization as a fundamental aspect of any successful transitional policy process from military to civilian life (see McGarry, 2010). If understood commensurate with desistance work for example (see inter alia Albertson, Irving, & Best, 2016), policy and research relating to the issues raised throughout this article may instead wish to consider the “institutionalized conduct” (Berger & Luckman, 1966) of service personnel, complete with their military “dependencies” and “idiosyncrasies” (Wainwright et al., 2016), as issues requiring resocialization *from* the military institution, rather than being reconstituted *within* civilian life. More careful consideration is needed when devising polices which propel people who may, or may not, have faced difficulties caused or exacerbated by service life back into civilian environments that champion and reinvent military culture.
